# Strategies to control HIV and HCV in methadone maintenance treatment in Guangdong Province, China: a system dynamic modeling study

**DOI:** 10.1186/s13011-017-0140-3

**Published:** 2018-01-10

**Authors:** Xia Zou, Yong Xu, Wen Chen, Yinghua Xia, Yin Liu, Cheng Gong, Li Ling

**Affiliations:** 1 Faculty of Medical Statistics and Epidemiology, School of Public Health, Sun Yat-sen University, Guangzhou, People’s Republic of China; 2 Sun Yat-sen Center for Migrant Health Policy, Sun Yat-sen University, Guangzhou, People’s Republic of China

**Keywords:** Methadone maintenance treatment, Human immunodeficiency virus, Hepatitis C, System dynamics

## Abstract

**Background:**

Human immunodeficiency virus (HIV) and hepatitis C virus (HCV) infections among methadone maintenance treatment (MMT) participants remain high. Optimized HIV and HCV prevention strategies for MMT clinics in resource-limited regions are urgently needed. This study aims to develop an MMT system dynamic model (SDM) to compare and optimize HIV and HCV control strategies in the MMT system.

**Methods:**

We developed an MMT-SDM structure based on literature reviews. Model parameters were estimated from a cohort study, cross-sectional surveys and literature reviews. We further calibrated model outputs to historical data of HIV and HCV prevalence among MMT participants in 13 MMT clinics of Guangdong Province. Lastly, we simulated the impact of integrated interventions on HIV and HCV incidence among MMT participants using the MMT-SDM.

**Results:**

The MMT-SDM comprises MMT clinics, MMT participants, detoxification centers, and HIV and HCV transmission, testing and treatment systems. We determined that condom promotion was the most effective way to reduce HIV infection (2013-2020: 2.86% to 1.76%) in MMT setting, followed by needle exchange program (2013-2020: 2.86% to 2.56%), psychological counseling (2013-2020: 2.86% to 2.71%) and contingency management (2013-2020: 2.86% to 2.72%). Health education had marginal impact on reducing HIV incidence among MMT participants (2013-2020:2.86% to 2.84%) from 2013 to 2020. By contrast, psychological counseling (2013-2020: 7.54% to 2.42%) and contingency management (2013-2020: 7.54% to 2.96%) had been shown to be the most effective interventions to reduce HCV incidence among MMT participants, followed by needle exchange program (2013-2020: 7.54% to 5.76%), health education (2013-2020: 7.54% to 6.35%), and condom promotion program (2013-2020: 7.54% to 6.40%). Notably, HCV treatment reduced HCV incidence by 0.32% (2013-2020: 7.54% to 7.22%).

**Conclusions:**

In conclusion, we generated a valuable system dynamic model to analyze the Chinese MMT system and to guide the decision-making process to further improve this system. This study underscores the importance of promoting condom use in MMT clinics and integrating psychosocial interventions to reduce HIV and HCV infections in MMT clinics in China.

**Electronic supplementary material:**

The online version of this article (10.1186/s13011-017-0140-3) contains supplementary material, which is available to authorized users.

## Background

Human immunodeficiency virus (HIV) and hepatitis C virus (HCV) have been the most prevalent infections among drug users around the world for several decades, and the epidemics remain severe today. Among the drug users, people who inject drugs are the most-at-risk population of HIV and HCV infections. The global HIV and HCV prevalence among people who inject drugs are estimated to be 17.8%, and 52.3%, respectively [1–3]. China has the largest number of drug users who are at high risk of HIV/HCV infection [4]. There were about 2.5 million known drug users in China in 2016, and among them, it was estimated 6.3% were infected with HIV and over 60% with HCV [5–7].

Methadone maintenance treatment (MMT) is widely accepted as an effective harm reduction program that lowers heroin cravings and reduces HIV/HCV-related risk behaviors among drug users [8], but the effectiveness of MMT on reducing HIV and HCV infections may vary in different regions [9]. Studies show that HIV and HCV incidence among drug users receiving MMT in China remains high [10]. This may largely due to the difference of mode in providing MMT in China. Most MMT clinics in China provide only simple services, including methadone dosing, urine monitoring, risk behavior follow-up and HIV/HCV testing. The simple services without effective interventions may limit the effectiveness of the MMT program [11]. Interventions such as health education, condom promotion and needle exchange programs have been implemented to curb the HIV/HCV epidemics among drug users in different settings [12–15]. Additionally, these measures have been shown to have various efficacies among different groups of at-risk populations. But these interventions were not routinely implemented in MMT program in China, and the HIV/HCV-lowering effects of integrating these services in MMT clinics are unknown.

The MMT program is a complex system involving multiple sectors and variables. The success of HIV and HCV prevention interventions is associated with the individuals’ behaviors and the performance of MMT clinics, police sectors and detoxification centers. Multiple causal loops and diagrams can be used to estimate the HIV and HCV prevalence and incidence among MMT participants. Most of the current mathematical models simulate HIV and HCV transmission, and the development of HIV and HCV prevention strategies has been largely targeting the transmission process guided by these models [16, 17]. However, a comprehensive analysis of the multi-sector factors, the associations between them and their contribution to the high HIV and HCV incidence among MMT participants has to our knowledge not yet been done.

Complex society systems can be modeled by system dynamic models in which causal diagrams are used to qualitatively describe the system and to quantify the relationships between factors based on differential equations [18]. These have been widely applied in various public health fields, including disease transmission, substance abuse and health care delivery [19]. HIV/AIDS system dynamic models have been developed. The system modeled multiple stages from susceptible, HIV stages one to three, early AIDS, late AIDS and cumulative deaths, and was used for epidemic projections and for simulating the effectiveness of interventions [20, 21]. A system dynamic model encompassing policy, organizational and individual factors has also been developed to determine the effectiveness of condom use programs on HIV and other sexually transmitted diseases, focusing mainly on female sex workers [22]. The impact of needle exchange programs and antiretroviral therapy on HIV and other transmitted diseases have similarly been modeled [20]. In addition, system dynamic modeling can be used to simulate the enrollment and drop-out of participants in a program [23]. Nevertheless, a system dynamic model that simulates the MMT system and evaluates the effectiveness of HIV and HCV prevention programs in the MMT system has not been developed. To fill this gap, we developed an MMT system dynamic model (MMT-SDM), which revealed the complexity of the MMT system in China, and used this model to explore the effectiveness of integrated HIV/HCV prevention programs in the MMT setting in reducing HIV and HCV infections.

## Methods

### MMT system dynamic model (MMT-SDM)

**Fig. 1:**
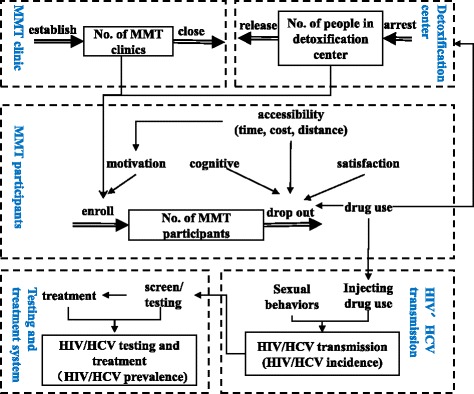
Framework of the MMT system in China

A system dynamic model is built based on a qualitative description of the model and quantitative equations that describe the relationships between factors. The model structure was designed by our group based on the available literature. The MMT-SDM comprises MMT clinics, MMT participants and detoxification centers and encompasses HIV and HCV transmission, testing and treatment. We decided the core elements of this model as number of MMT clinics, number of people in detoxification center, number of MMT participants, HIV and HCV prevalence and incidence (Fig. 1). We then individually counseled the physicians and nurses in MMT clinics to develop the causal diagram within each sub-system.

The main indicators of our research are HIV and HCV prevalence and incidence among MMT participants, which were determined by the number of MMT participants and the number of HIV and HCV infections. Re-enrollments and drop-outs in the MMT clinics change the number of MMT participants in a given time frame [24]. The number of enrolled MMT participants has been found to associate with the establishment of new MMT clinics and the motivation of MMT participants. Some MMT participants who were previously arrested and have since been released from the detoxification center might get reenrolled in MMT [25]. Frequent enrollments and drop-outs are common for MMT participants. The main reasons for drop-out are concurrent drug use and accessibility to, perception of and satisfaction with the MMT [8]. In addition, a subset of MMT participants might be arrested due to illicit drug use and sent to detoxification centers [25].

High-risk behaviors occurred among a high proportion of MMT participants. Unprotected sexual intercourse, drug use and injection drug use among MMT participants accelerate the transmission of HIV/HCV and increase the incidence and prevalence of HIV/HCV among MMT participants. All MMT participants are tested for HIV/HCV annually in MMT clinics, and those with positive results are referred to the center for disease control and prevention for confirmation. Only a small percentage of MMT participants will then go on to receive HIV or HCV treatment [26]. Causal diagrams of the MMT-SDM are described based on literature review (Additional file 1: Figure S1a-d).

### Parameters and data sources

The parameters of the model were collected from a cohort study, a cross-sectional study, and literature review and meta-analysis (Additional file 1: Table S1). We established a cohort that included all participants of 13 MMT clinics in Guangdong Province, China, from 2006 to 2013. We collected information on the MMT clinics (number of MMT participants, number of MMT clinics, new entrants to MMT, etc.) and the participants (daily doses, drug use, injection drug use, needle sharing behaviors, HIV/HCV testing and results, etc.). Between December 2011 and January 2012, a cross-sectional survey was conducted in 13 MMT clinics in Guangdong Province. We investigated the motivation and detoxification intent of MMT participants, the treatment time, cost and distance to the MMT clinics, the satisfaction of participants, and the associations of these factors. The design of this survey has been reported elsewhere [27]. For factors that could not be acquired from the cohort and cross-sectional studies, we estimated the parameters based on available systematic reviews and meta-analyses, single and multiple papers, and the consultation with doctors in MMT clinics.

### System dynamic equations

System dynamic equations consist of stock, flow, auxiliary, and constant variables. The reference ranges of the constants can be determined from surveys and published reports (Additional file 1: Table S2). Stock equations express the accumulation of stock variables over time. Flow equations express the change of variables over time. Auxiliary equations express the relationship between two factors. We constructed auxiliary equations based on analyses of data from cohort study or cross-sectional survey, and published literatures (Additional file 1: Table S2).

### Model calibration

The model was calibrated using HIV and HCV prevalence data collected in 13 MMT clinics in Guangdong Province from 2006 to 2013. We calculated the output values of the model and compared output values with the historical data and the confidence intervals.

### Scenario analysis

We estimated the effectiveness of interventions to reduce misconceptions about MMT, unprotected sexual intercourse, drug use, injection drug use and needle sharing behaviors in China and to improve the proportion of ART and HCV treatment based on literature review (Additional file 1: Table S3). We simulated how these interventions affect HIV and HCV incidence among MMT participants using the MMT-SDM.

The model was constructed using Vensim DSS for Windows Version 5.6a (Copyright 1988-2006, Ventana Systems, Inc.). HIV and HCV simulation results were compared with historical data using R version 3.3.1.

## Results

### Simulations of HIV and HCV prevalence among MMT participants

**Fig. 2:**
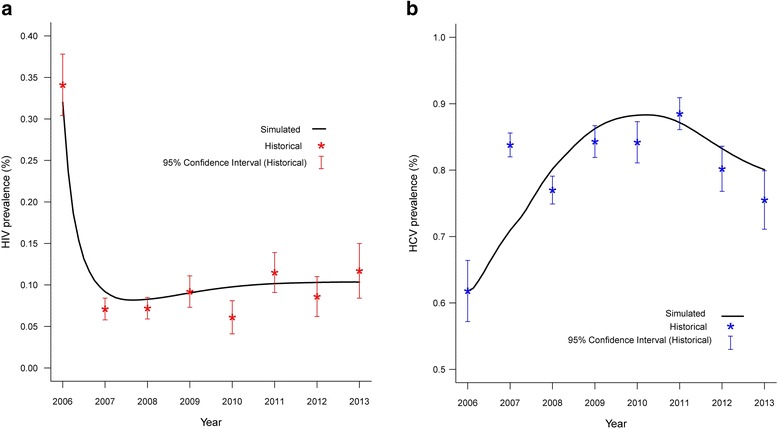
Fitness of the simulated numbers compared to the historical HIV (**a**) and HCV (**b**) prevalence among MMT participants. (Note: number of MMT participants were 605, 2055, 2864, 2942, 2710, 3301, 3368, 2840 in 2006-2013)

The number of MMT participants in 13 MMT clinics were ranged from 605 to 3368 in 2006 to 2013. The MMT-SDM was calibrated using historical data on HIV and HCV prevalence among MMT participants. The simulated data on HIV prevalence among MMT participants were 34.1% to 11.7% in 2006-2013, while the HCV prevalence in 2006-2013 were 61.8%~88.5%. [28]. From 2006 to 2013, most of the simulated HIV prevalence among MMT participants fell within the 95% confidence interval of the historical data, except for the prevalence in 2007 and 2010. Meanwhile, the historical HCV prevalence among MMT participants was higher than the simulated numbers in 2007, and lower in 2008 and 2010. Other values of the simulated HCV prevalence of the model fell within the 95% confidence interval of the historical data values (Fig. 2).

### Projection of HIV and HCV incidence among MMT participants in 2006-2020

**Fig. 3:**
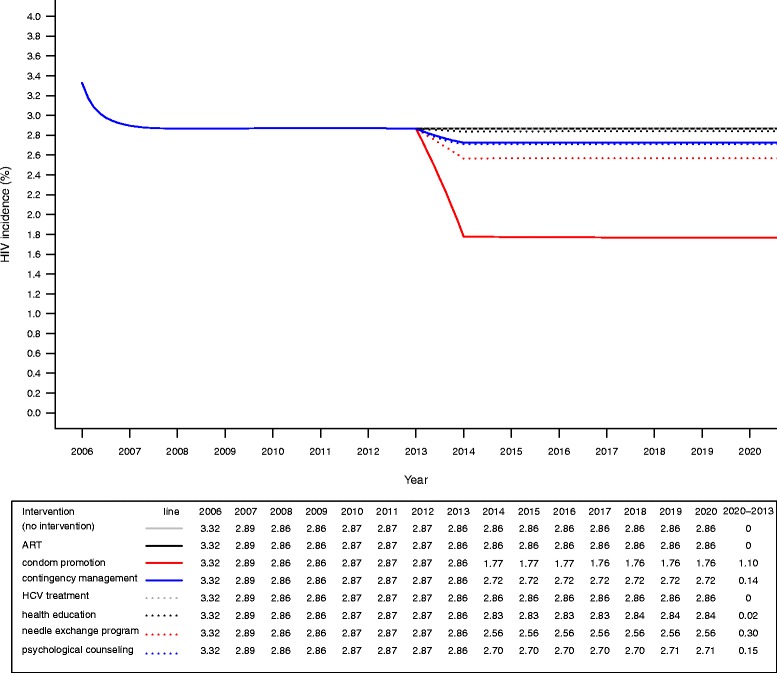
Effectiveness of integrated interventions on reducing HIV incidence among participants in MMT

**Fig. 4:**
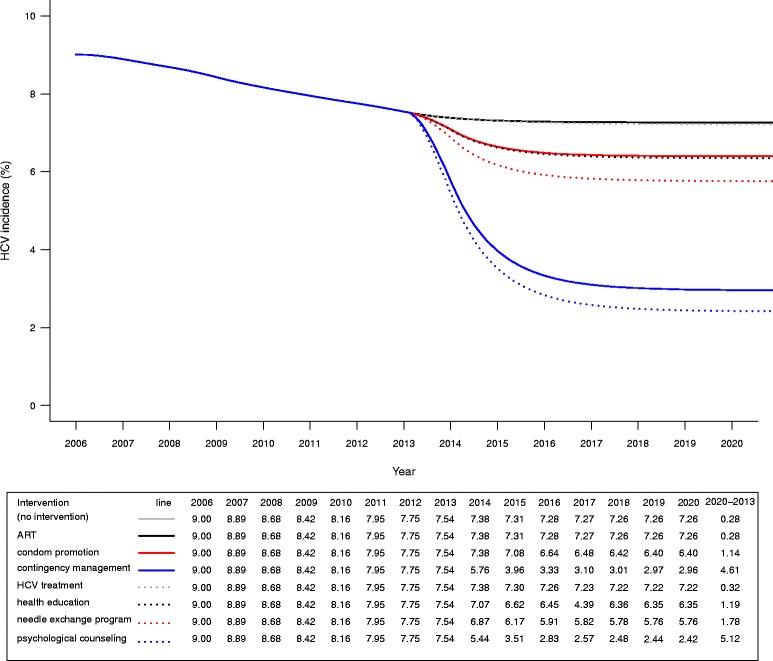
Effectiveness of integrated interventions on reducing HCV incidence among participants in MMT

In current scenario, we estimated that the HIV incidence among MMT participants lowered from 3.32% to 2.86% in 2006-2013, then was stable at 2.86% in 2014-2020 (Fig. 3). HCV incidence among MMT participants substantially reduced from 9.00% to 7.54% in 2006-2013, and continued to reduce from 7.38% to 7.26% in 2014-2020 (Fig. 4).

### Effects of interventions on HIV and HCV incidence among MMT participants

Condom promotion was found to be the most effective way to reduce HIV infection (2013-2020: 2.86% to 1.76%) in MMT setting, followed by needle exchange program (2013-2020: 2.86%, psychological counseling (2013-2020: 2.86% to 2.71%) and contingency management (2013-2020: 2.86% to 2.72%). From 2013 to 2020, HIV incidence among MMT participants was projected to decrease 1.1%, 0.30%, 0.15% and 0.14% by implementation of condom promotion, needle exchange program, psychological counseling and contingency management, respectively. Health education had marginal impact on reducing HIV incidence among MMT participants (2013-2020:2.86% to 2.84%), with only 0.02% of reduction from 2013 to 2020 (Fig. 3).

By contrast, psychological counseling (2013-2020: 7.54%-2.42%) and contingency management (2013-2020: 7.54%-2.96%) had been shown to be the most effective interventions to reduce HCV incidence among MMT participants. The HCV incidence reduced 5.12% and 4.61% for psychological counseling and contingency management, respectively. While the HCV incidence was 1.78% lower with a needle exchange program (2013-2020: 7.54%-5.76%) than with no intervention (2013-2020: 7.54%-7.26%). Similarly, the HCV incidence decreased 1.19% and 1.14% for health education (2013-2020: 7.54%-6.35%) and condom promotion program (2013-2020: 7.54%-6.40%), respectively. Notably, HCV treatment reduced HCV incidence by 0.32% (2013-2020: 7.54%-7.22%) (Fig. 4).

## Discussion

We investigated the Chinese MMT system framework and created a MMT system dynamic model (MMT-SDM) to qualitatively describe the MMT system in China and to simulate the relationship between several factors of this system. Using this model, we determined that condom promotion programs in the MMT system were more effective in reducing HIV incidence than psychological counseling, contingency management, needle exchange programs and health education. While psychological counseling and contingency management are more effective than other interventions in reducing HCV incidence. By contrast, ART and HCV treatment had only limited impact on the HIV and HCV incidence in MMT settings.

An earlier study developed a dynamic system to simulate the enrollment and drop-out of participants in a HIV intervention program [23]. This model provided valuable information on enrollment and drop-out processes and on the interaction of factors in the system. Based on this insight, we integrated the functions of detoxification centers, cost, time and distance into our model. The MMT-SDM was validated using survey data and calibrated to fit the historical data well. Although there were outliers in a few years, these values were within the upper or lower 25% of the historical values. In real-world scenarios, HIV and HCV prevalence may be changed by many unexpected factors. Therefore, a small proportion of outliers are acceptable.

This study reveals disparity in efficacy of condom promotion program in reducing HIV and HCV incidence. Our results indicate that integrating a condom promotion program in MMT is more effective than other interventions. While condom program is only more effective than HCV treatment in reducing HCV incidence, and less effective than other interventions. The disparities may due to the difference transmission mode of HIV and HCV. The transmission of HIV mainly through risky sexual behaviors these years. Although the transmission efficacies of HIV through risky sexual behaviors are much lower than through injection behaviors, condom promotion programs target more MMT participants, and risky sexual behaviors among drug users on MMT are prevalent. A high proportion of unprotected sexual intercourse has been found among MMT participants [24]. By actively providing condoms in MMT clinics, the proportion of unprotected sexual behaviors can be largely reduced. By contrast, HCV was more likely to transmit through injecting behaviors. Therefore, condom promotion program may less effective than interventions that target at injecting behaviors, such as needle exchange program, psychological counseling, contingency management and health education.

Our findings show that psychological counseling is the most effective intervention in reducing HCV incidence. Psychological counseling has developed early and has been widely accepted as an effective behavior-changing strategy. Counseling has been applied in various settings, including in community-based MMT clinics. Numerous studies have shown it to be highly efficacious in reducing positive urine results and improving the retention of MMT participants [29–32]. Another study also explored the effect of psychological counseling on drug use and injection drug use behaviors [33]. Contingency management is another psychological intervention that has been shown to reduce drug use behaviors in MMT settings by targeting behavior changes. Therefore, improving retention among MMT participants and reducing HCV-related risk behaviors through psychological counseling and contingency management may effectively reduce the HCV incidence. But the effects of psychological counseling and contingency management in reducing HCV incidence are different. This could be explained by the differences in the approach to achieving behavior changes. The goal of psychological counseling is to change behavior through counseling and to ultimately achieve the psychological recovery of the participants. Contingency management, on the other hand, encourages participants to follow certain behaviors by enticing them with opportunities to win prizes, which contains an element of chance [34]. Therefore, psychological counseling may be more likely to achieve the psychological recovery of MMT participants.

Health education programs aim to change behaviors by improving the understanding of HIV and HCV transmission and by changing misconceptions. MMT participants are often poorly educated about MMT and believe that the MMT clinic is solely a place to get methadone, which may help them avoid heroin addiction. Most participants do not consider MMT clinics as the place to help them recover from drug abuse. These misconceptions may prevent them from changing HIV/HCV-related risk behaviors [35]. Health education geared toward improving the perception of MMT may thus help to promote the psychological recovery of MMT participants. However, it would be a long process to go from cognitive changes to behavioral changes through health education with persistent intervention.

Our finding demonstrates that integrating HCV treatment and ART in MMT clinics has marginal impact on reducing HCV incidence. Previous studies have demonstrated that ART can lower the viral load of AIDS patients, which will decrease the likelihood of transmission [36]. But it will be a very long process through lowering the transmission risks among MMT participants. No obvious short-term effect can be seen. In comparison, HCV is a curable disease. New HCV infections can be rapidly reduced by treating existing infections. Furthermore, the HCV prevalence may also be further decreased due to effective treatment, thus lowering the number of new infections. One challenge is that the effectiveness may be limited by low adherence to HCV treatment and by reinfection through injection behaviors [37]. The high cost of HCV treatment and prolonged treatment duration prevent drug users from receiving and retaining treatment [38]. In the past, HCV treatment required injections for at least 12 months, and a full 48-week course of HCV treatment cost approximately 9600 US dollars, which represents a high economic burden for drug users on MMT [39]. In 2016, the development of a new oral HCV therapy promises to greatly improve treatment adherence. This therapy has been approved for the third phase of clinical trials in China and is expected to benefit the HCV-infected population in the upcoming years [40].

This study provides a tool for monitoring the MMT system in China and for simulating the effect of interventions on HIV/HCV infection in the MMT system. By utilizing this tool, we recommend enhancing the psychological services in MMT clinics. Many countries have promoted comprehensive services in MMT clinics. In particular, Canada’s MMT guidelines propose that psychological counseling and contingency management should be the main domains of MMT services [41]. In contrast, the Chinese MMT guidelines provide only brief information on methadone intake and HIV/HCV testing. We strongly urge the government to draft a more detailed guide that integrates these comprehensive services in MMT clinics to improve the effectiveness of the MMT program in China. Additionally, condom promotion should be integrated in MMT clinics to prevent HIV/HCV from transmission through unprotected sexual behaviors, which are highly prevalent among MMT participants. MMT participants account for a large number of HIV and HCV infections, and MMT is a platform that can address these patients. Therefore, the government should fully utilize this platform for the prevention of HIV and HCV in China.

There are several limitations to this study that should be mentioned. Importantly, a multitude of parameters are modeled in this MMT-SDM, and since some parameters were sourced from a single study, there may be bias of the estimates of the parameters. Furthermore, the effectiveness of interventions used in this model was estimated based on the available literature, and there were variations between different studies. To minimize bias, we tried to use meta-analysis estimates of parameters and fit the model using real data. A long-term cohort should be established to confirm the incidence of HIV and HCV, and intervention pilot and follow-up study should be conducted to confirm the effectiveness and feasibility of the intervention in the real world.

## Conclusions

In conclusion, we generated a valuable system dynamic model to analyze the Chinese MMT system and to guide the decision-making process to further improve this system. This study underscores the importance of promoting condom use in MMT clinics and integrating psychosocial interventions to reduce HIV and HCV infections in MMT clinics in China.

## Additional file

Additional file 1: Figure S1a. Causal diagram of MMT clinics, participants and detoxification centers. **Figure S1b**. Causal diagram of HIV/HCV transmission. **Figure S1c**. HIV testing and treatment systems causal diagram. **Figure S1d**. Causal diagrams of HIV and HCV testing and treatment systems. **Figure S2a**. Stock and flow diagram of MMT clinics, participants and detoxification centers. **Figure S2b**. Stock and flow diagram of HIV and HCV testing and treatment systems. **Figure S2c**. Stock and flow diagram of HIV transmission system. **Figure S2d**. Stock and flow diagram of HCV transmission system. **Table S1**. Parameters of MMT dynamic model. **Table S2**. System dynamic equations of the MMT system dynamic model. **Table S3**. Effectiveness of health education, psychological counseling, contingency management, needle exchange program, condom promotion, ART and HCV treatment. (PDF 1349 kb)

## Abbreviations

HCV: Hepatitis C virus
HIV: Human immunodeficiency virus
MMT: Methadone maintenance treatment
SDM: System dynamic model
