# Supplementary material for: Strategies to control HIV and HCV in methadone maintenance treatment in Guangdong Province, China: a system dynamic modeling study
Source: Subst Abuse Treat Prev Policy. 2018 Jan 10;13:1. doi: 10.1186/s13011-017-0140-3 (PMC5763976; doi:10.1186/s13011-017-0140-3)
Supplement: Additional file 1: Figure S1a. — Causal diagram of MMT clinics, participants and detoxification centers. Figure S1b. Causal diagram of HIV/HCV transmission. Figure S1c. HIV testing and treatment systems causal diagram. Figure S1d. Causal diagrams of HIV and HCV testing and treatment systems. Figure S2a. Stock and flow diagram of MMT clinics, participants and detoxification centers. Figure S2b. Stock and flow diagram of HIV and HCV testing and treatment systems. Figure S2c. Stock and flow diagram of HIV transmission system. Figure S2d. Stock and flow diagram of HCV transmission system. Table S1. Parameters of MMT dynamic model. Table S2. System dynamic equations of the MMT system dynamic model. Table S3. Effectiveness of health education, psychological counseling, contingency management, needle exchange program, condom promotion, ART and HCV treatment. (PDF 1349 kb) [file 13011_2017_140_MOESM1_ESM.pdf]

Supplementary material

1. Descriptions of causal loops of the MMT system

Causal loop diagrams describe the relationships and feedback loops of the factors in a complex dynamic system. The causal loops diagram consists of positive or negative feedback loops or chains.

(1) Description of MMT clinics, detoxification centers and MMT system participants

The establishment of new MMT clinics was one of the most important factors that decided the number of new MMT participants. Drug users with the intent and motivation for treatment would participate in the MMT program, thus increasing the number of MMT participants. MMT participants tend to be less satisfied as the number of people on MMT increase, resulting in drop-outs and decreased numbers of MMT participants. Recycling forms a negative causal loop (B) that decreases the numbers of MMT participants whereas drop-outs may be re-enrolled in the MMT program after a certain period of time (average of about 6 months) [1]. This positive causal loop also increases the number of MMT participants (R1). Other reasons that may cause participants to drop out of the MMT system in China include time, cost, distance to the MMT clinic, misconceptions about MMT, doses and concurrent drug use behaviors [2-4]. MMT participants use drugs and drop out of the program, and several studies have documented the phenomenon of arrests of drug users who are on MMT [5, 6]. Those who are arrested are required to enter a detoxification center and are released after two years of detoxification. Some of them re-enroll in MMT through the referral of the center (R2). These causal loops describe the dynamics of the participants in the MMT program (Figure S1a).

域代码已更改

域代码已更改

域代码已更改

域代码已更改

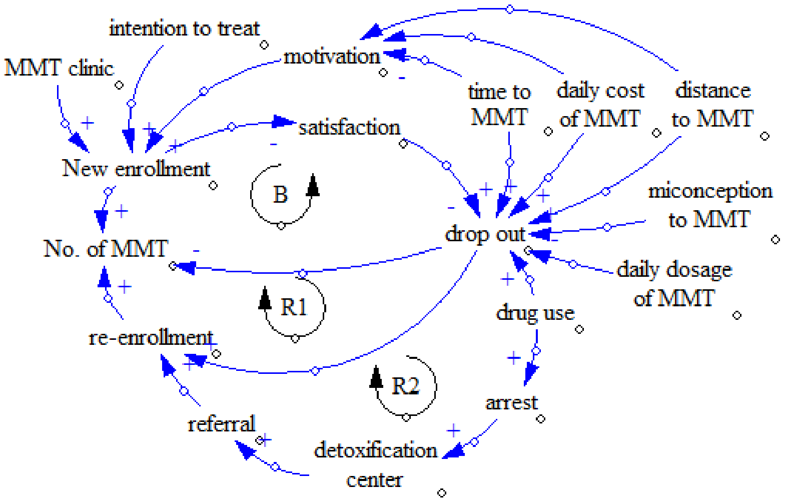

Figure S1a. Causal diagram of MMT clinics, participants and detoxification centers.

(2) Description of HIV and HCV transmission systems

The HIV and HCV transmission models simulate HIV and HCV transmission via both unprotected or protected sexual intercourse and via sterilized or unsterilized needles/syringes (Figure S1b).

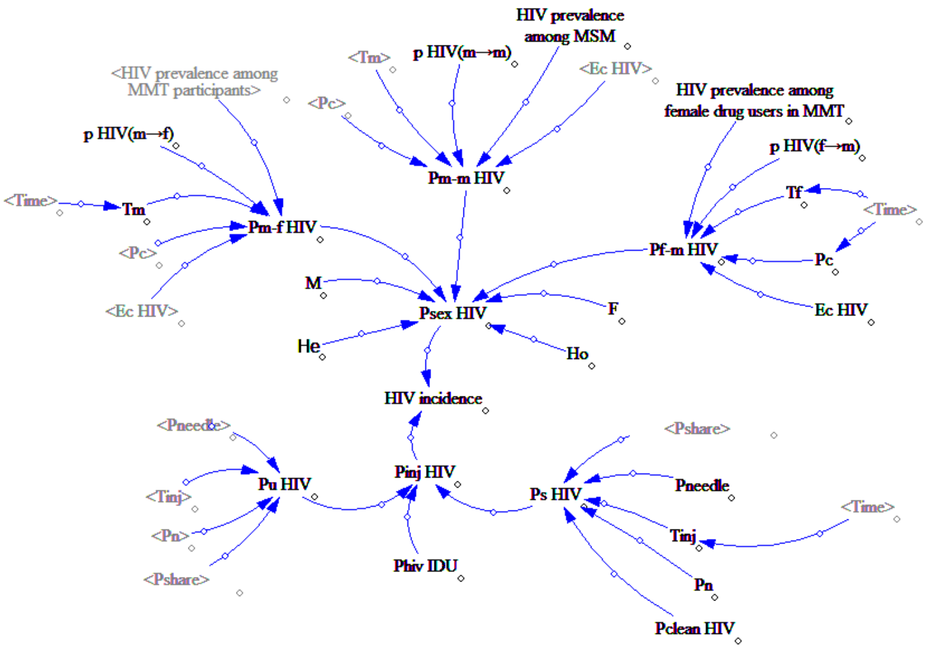

Figure S1b. Causal diagram of HIV/HCV transmission.

The probability of HIV transmission from a positive female to a negative male through sexual intercourse is:

$$p_{f-m}HIV = \left\{1 - \left(1 - p_{HIV\,(f\to m)}\right)^{T_f \times P_c} \times \left(1 - (1 - E_c HIV) \times p_{(f\to m)}\right)^{T_f \times P_c}\right\} \times HIV\ prevalence\ among\ female\ drug\ users\ in\ MMT$$

While the probability of HIV transmission from a positive male to a negative female through sexual intercourse is:

$$p_{m-f}HIV = \left\{1 - \left(1 - p_{HIV\,(m\to f)}\right)^{T_m \times P_c} \times \left(1 - \left(1 - E_{c\,HIV}\right) \times p_{(m\to f)}\right)^{T_m \times P_c}\right\} \times HIV\ prevalence\ among\ MMT\ participants$$

The probability of HIV transmission from a positive male to a negative male through sexual intercourse is:

$$p_{m-m}HIV = \left\{1 - \left(1 - p_{HIV\,(m\to m)}\right)^{T_m \times P_c} \times \left(1 - \left(1 - E_{c_{HIV}}\right) \times p_{(m\to m)}\right)^{T_m \times P_c}\right\} \times HIV\ prevalence\ among\ MSM$$

$p\ HIV_{(f\to m)}$: the infectivity of HIV from a positive female to a negative male;

T_f: the number of sexual encounters among female drug users each year;

P_c: the proportion of unprotected sexual intercourse;

E_c HIV: the effectiveness of condoms in protecting from HIV/HCV transmission;

$p\ HIV_{(m\to f)}$: the infectivity of HIV/HCV from a positive male to a negative female;

T_m: the number of sexual encounters among male drug users each year;

Then, the probability of HIV/HCV transmission through sexual behaviors is:

$$P_{sex}HIV = P_{m-f}HIV \times F \times H_e + P_{m-m}HIV \times M \times H_o + P_{f-m}HIV \times M$$

F: the proportion of females on MMT;

M: the proportion of males on MMT;

He: the proportion of heterosexuality among male participants;

Ho: the proportion of homosexuality among male participants;

The probability of HIV/HCV transmission through sterilized needles/syringes is:

$$P_sHIV = 1 - [1 - P_{needle} \times (1 - P_{clean}HIV)]^{P_n \times T_{inj} \times P_{share}}$$

While the probability of HIV/HCV transmission through unsterilized needle/syringes is:

$$P_uHIV = 1 - [1 - P_{needle}]^{(1-P_n) \times T_{inj} \times P_{share}}$$

P_{needle}: the probability of HIV/HCV transmission per injection;

$P_{clean}\,HIV$: the probability of successful sterilization;

P_n: the proportion of sterilized needles/syringes;

T_{inj}: the number of injections each year;

P_{share}: the proportion of shared needles/syringes.

Then, the probability of HIV/HCV transmission through injection behaviors is:

$$P_{inj}HIV = [1 - (1 - P_s\ HIV) \times (1 - P_u\ HIV)] \times P_{hiv}\ IDU$$

$P_{hiv\ IDU}$: the prevalence of HIV/HCV among injection drug users.

The HIV/HCV incidence is therefore calculated as: $HIV\ incidence = 1-(1-P_{sex}\ HIV)\times(1-P_{inj}\ HIV)$.

The HCV transmission process is similar with HIV transmission process.

(3) Description of testing and treatment systems

The HIV/HCV testing and treatment systems describe the progression from undiagnosed to treatment or death. HIV-infected MMT participants receive testing in MMT clinics every year. Some of the diagnosed HIV-infected individuals receive treatment. However, the proportion of drop-outs among antiretroviral therapy patients who are receiving MMT is high. A proportion of the patients die after a period of time (Figure S1c). HCV-infected MMT participants undergo a similar process including undiagnosed, diagnosed and on treatment. Unlike HIV, HCV can be cured after completion of treatment, and a small percentage of infections can be spontaneously cleared by the human immune system (Figure S1d).

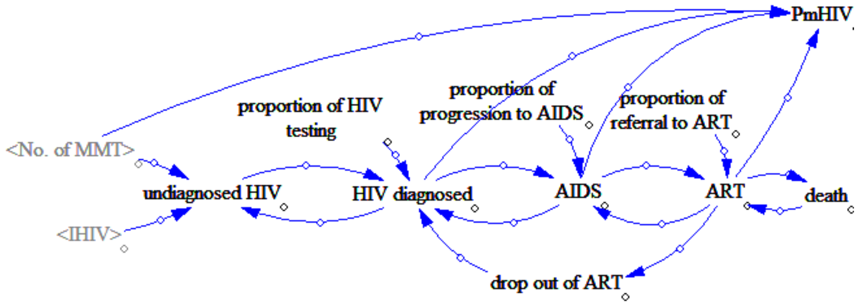

Figure S1c. HIV testing and treatment systems causal diagram

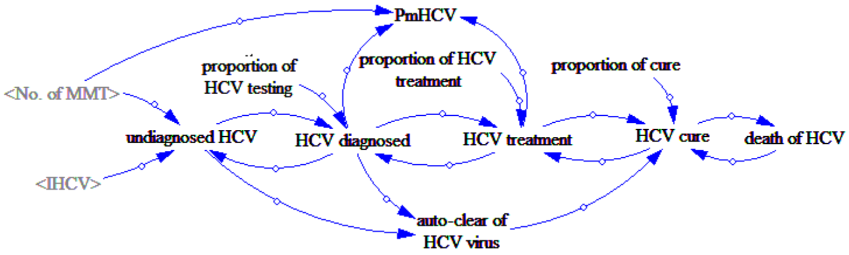

Figure S1d. Causal diagrams of HIV and HCV testing and treatment systems.

2. Stock and flow diagram of MMT-SDM

Based on the causal loops, we further created stock and flow diagram of MMT-SDM. A rectangle represents the stock in system dynamics model. We use black arrows to indicate the inflow in and outflow of the stock variable. Blue arrows are showing the causal relations between auxiliary variables or impact of constant variable on auxiliary/flow variables.

In this model, “MMT CLINIC”, “MMT RETENTION”, “DC CENTER”, “UNDISAGNOSED HIV/HCV”, “DIAGNOSED HIV/HCV”, “AIDS”, “ART”, “CURE FOR HCV”, and “HCV CLEARANCE” are stock variables that represent the numbers of individuals stocked at this stage in system dynamic model. Flow variables (new enrollment, re-enrollment etc.) indicate the change of number of individuals within a certain time limit. Auxiliary variables are mediate variables, which express the information transformation between flows and stocks. Constant variables are constant within the study period (Figure S2a-d).

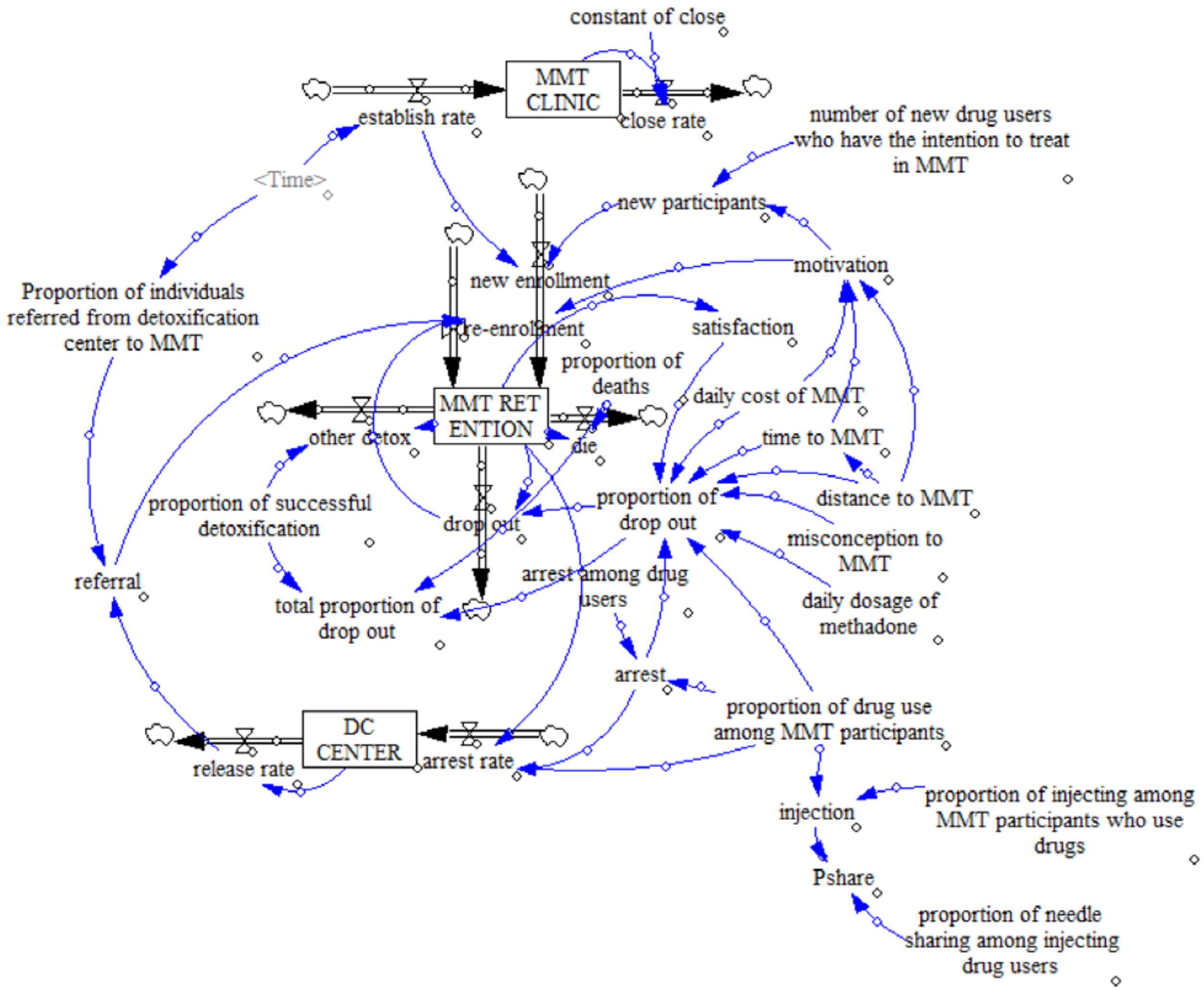

Figure S2a. Stock and flow diagram of MMT clinics, participants and detoxification centers.

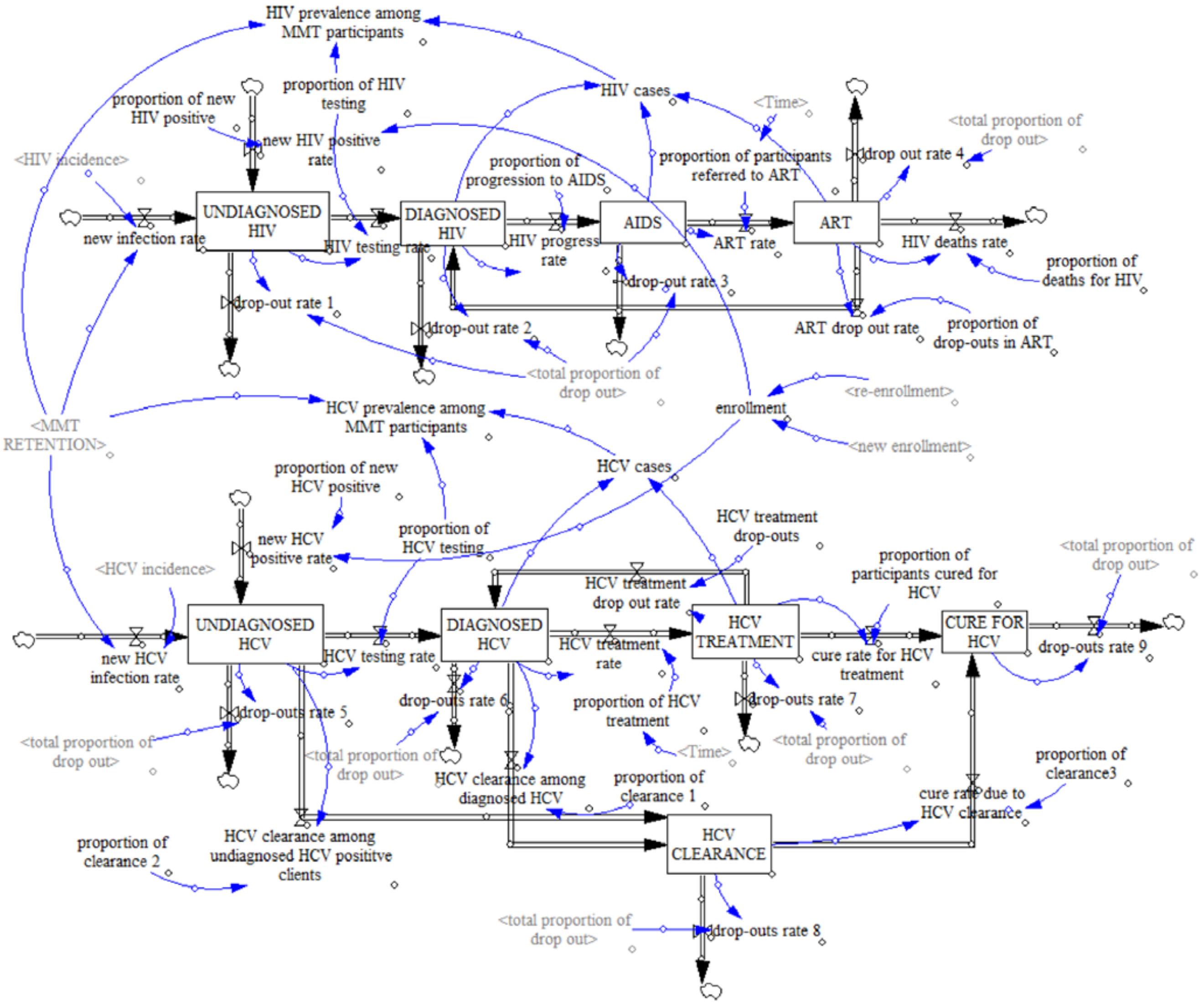

Figure S2b. Stock and flow diagram of HIV and HCV testing and treatment systems.

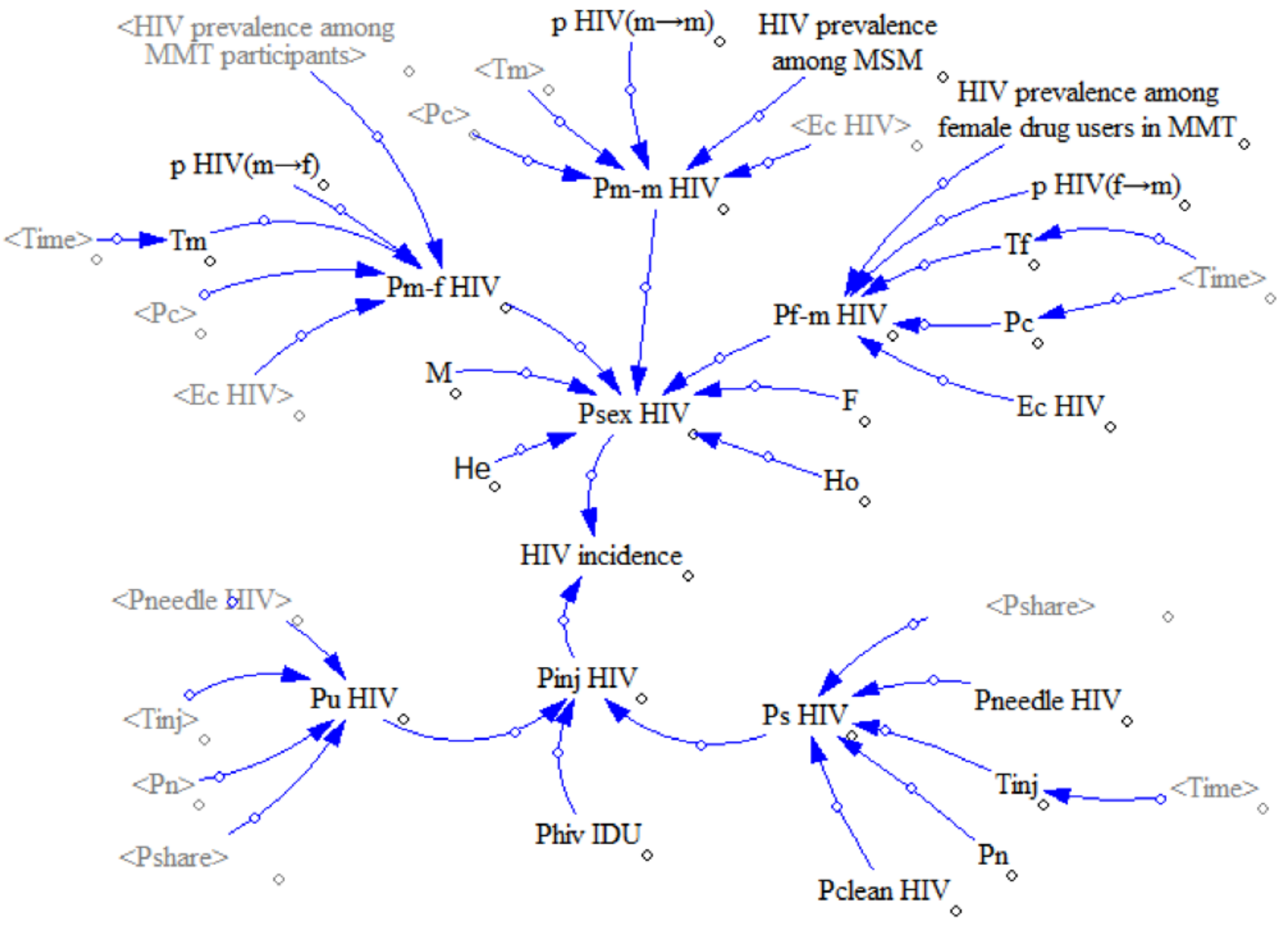

Figure S2c. Stock and flow diagram of HIV transmission system.

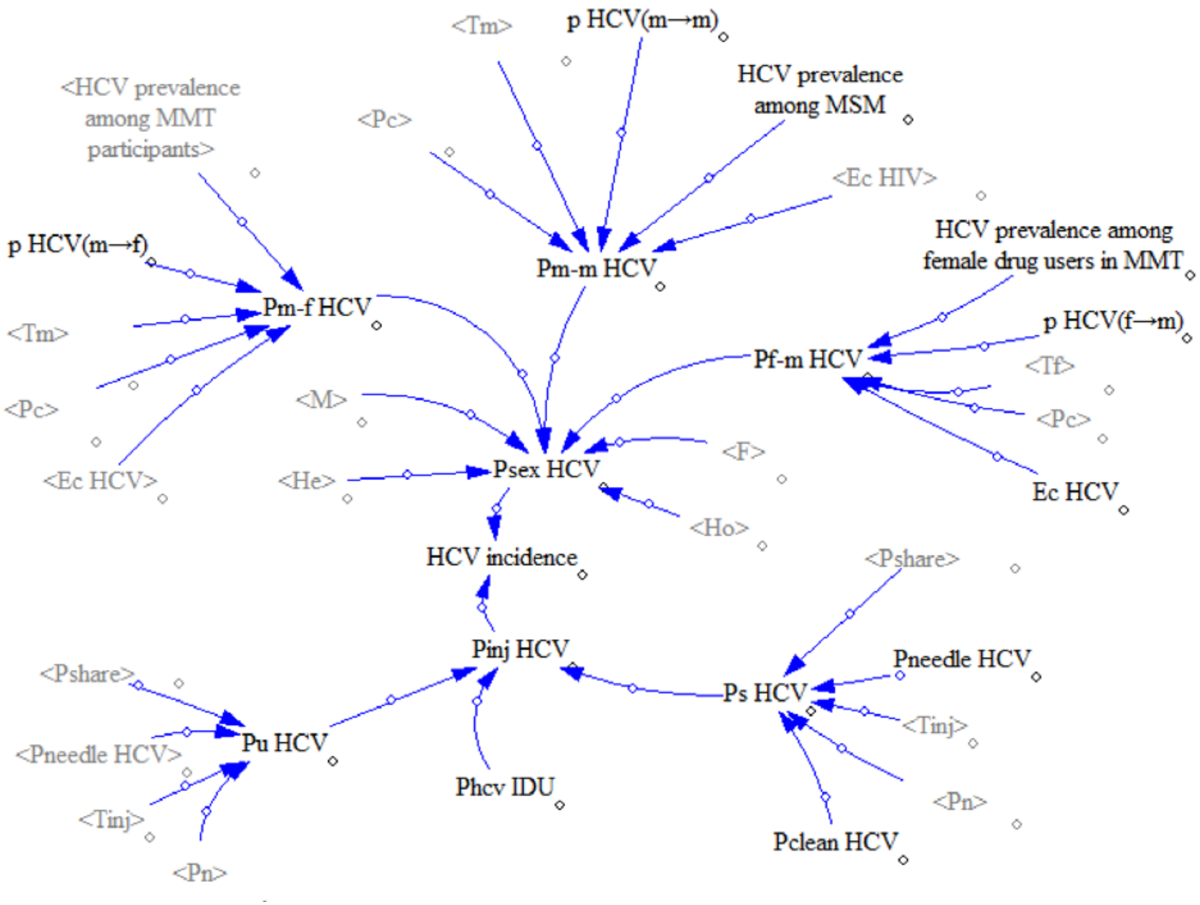

Figure S2d. Stock and flow diagram of HCV transmission system.

3. Parameter collection

Table S1 parameters of MMT dynamic model

No	Parameter in model	Description	Estimates	Lower value	Higher value	Sources/References
1	daily cost of MMT	daily cost of MMT (RMB)	20	15	25	CS[2]
2	distance to MMT	distance to MMT (kilomiter)	5	3.75	6.25	CS[2]
3	Misconception_	misconception (%)	~~0.~~98	~~0.~~50.6	~~0.~~98.2	CS[3]
4	daily dosage of methadone	daily dosage of methadone (ml)	50	28.5	74.7	CS,literatures [7, 8]
5	proportion of injection among MMT participants who use drugs	proportion of injection among MMT participants who use drugs (%)	~~0.~~60	~~0.~~49	~~0.~~80	Chort,Meta [9, 10]
6	proportion of drug use among MMT participants	proportion of drug use among MMT participants (%)	~~0.~~20	~~0.~~15.7	~~0.~~33.5	Cohort,Meta[9-11]
7	proportion of needle sharing among injecting drug users	proportion of needle sharing among injecting drug users (%)	~~0.~~30	~~0.~~25	~~0.~~35	Cohort,Meta [9]
8	proportion of successful detoxification	proportion of successful detoxification (%)	~~0.0~~0.1	0.~~00~~1	0.~~00~~3	Literatures [12]

9	number of new drug users who have the intention to treat in MMT	number of new drug users who have the intention to treat in MMT	600	450	750	consult
10	arrest among drug users	proportion of drug users being arrested (%)	~~0.~~20	~~0.~~15	~~0.~~25	Meta[11]
11	p~~P~~roportion of individuals referred from detoxification center to MMT	~~P~~proportion of individuals referred from detoxification center to MMT (%)	~~0.~~20	~~0.~~15	~~0.~~26	literatures,consult [13, 14]
12	p HIV(m→f)	HIV infectivity from positive male to negative female	0.00124	0.0001	0.0014	literatures[15]
13	p HIV(f→m)	HIV infectivity from positive female to negative male	0.00377	0.0013	0.011	literatures[15]
14	p HIV(m→m)	HIV infectivity from positive male to negative male	0.014	0.002	0.025	literature[16]
15	p HCV(m→f)	HCV infectivity from positive male to negative female	0.00125	0	0.03	literatures [17, 18]
16	p HCV(f→m)	HCV infectivity from positive female to negative male	0.00125	0	0.03	literatures [17, 18]
17	p HCV(m→m)	HCV infectivity from positive male to negative male	0.0014	0	0.066	literatures[19]
18	Tm	the number of sexual intercourses among male drug users each year	180	120	240	literatures [20]

19	Tf	the number of sexual intercourses among female drug users each year	120	36	288	literatures [21]
20	HIV prevalence among female drug users in MMT	HIV prevalence among female drug users in MMT (%)	~~0.0~~8	~~0.0~~1.6	~~0.0~~8.3	literatures [22, 23]
21	HIV prevalence among MSM	HIV prevalence among MSM (%)	~~0.~~11.6	~~0.0~~8	~~0.~~16.6	literature[24]
22	HCV prevalence among female drug users in MMT	HCV prevalence among female drug users in MMT (%)	~~0.0~~1.6	~~0.0~~1.2	~~0.0~~2.0	literatures [25]
23	HCV prevalence among MSM	HCV prevalence among MSM (%)	~~0.0~~1.2	~~0.0~~1.0	~~0.0~~1.6	literatures[19]
24	Pc	the proportion of unprotected sexual intercourses (%)	~~0.~~75	~~0.~~70	~~0.~~80	Meta[9]
25	Ec HIV	the effectiveness of condom in protecting HIV transmission (%)	~~0.~~80	~~0.~~70	~~0.~~95	literatures [26-28]
26	Ec HCV	the effectiveness of condom in protecting HCV transmission (%)	~~0.~~80	~~0.~~70	~~0.~~95	literatures [26-28]
27	M	the proportion of male MMT participants (%)	~~0.~~90	~~0.~~76	~~0.~~97	CS
28	F	The proportion of female MMT participants (%)	~~0.~~10	~~0.0~~3	~~0.~~24	CS
29	Pneedle HIV	the infectivity of HIV when sharing injecting drugs	0.01	0.0063	0.024	literatures [29]

30	Pneedle HCV	the infectivity of HCV when sharing injecting drugs	0.03	0.015	0.04	literatures [30]
31	Tinj	times of injection each year	80	73	105	literatures [9]
32	Pshare	the proportion of sterilized needles/syringes (%)	~~0.~~40	~~0.~~10	~~0.~~50	literatures [31, 32]
33	Pclean HIV	the probability of successful sterilization for HIV (%)	~~0.~~70	~~0.~~60	~~0.~~75	literatures [33]
34	Pclean HCV	the probability of successful sterilization for HCV (%)	~~0.~~35	~~0.~~30	~~0.~~40	literatures [34, 35]
35	Phiv IDU	the prevalence of HIV among injecting drug users (%)	~~0.~~10	~~0.0~~9	~~0.~~14	literatures [36]
36	Phcv IDU	the prevalence of HCV among injecting drug users (%)	~~0.~~80	~~0.~~50	~~0.~~90	literatures [37]
37	proportion of clearance	the clearance rate of HCV (%)	~~0.~~13	0	~~0.~~80	literatures [38]
38	proportion of HCV treatment	the proportion of MMT participants on HCV treatment (%)	~~0.~~40	~~0.~~20	~~0.~~60	consult
39	HCV treatment drop-outs	the proportion of drop-out among MMT participants who are on HCV treatment (%)	~~0.~~24	~~0.~~10.3	~~0.~~29.2	literatures [39, 40]
40	proportion of cure for HCV	the proportion of recovery after HCV treatment (%)	~~0.~~62	~~0.~~25	~~0.~~87	literatures[40, 41]

41	proportion of HCV testing	proportion of HCV testing among MMT participants (%)	~~0.~~800	~~0.~~78	~~0.~~82	cohort, literatures [42]
42	proportion of HCV testing	proportion of HIV testing among MMT participants (%)	~~0.~~800	~~0.~~78	~~0.~~82	cohort, literatures [42]
43	proportion of new HIV positive	proportion of new HIV infected MMT new entrants (%)	~~0.~~10.8	0.0~~0~~	~~0.~~24.7	literatures [23]
44	ART drop out rate	proportion of drop out among MMT participants on ART (%)	~~0.~~25	~~0.~~17	~~0.~~29	literatures [43]
45	proportion of participants referred to ART	proportion of MMT participants who referred to ART among those who are diagnosed of AIDS (%)	~~0.~~4.0	~~0.0~~1.0	~~0.~~65.0	literatures [44]
46	proportion of progression to AIDS	proportion of HIV progress to AIDS (%)	~~0.~~25	~~0.~~22.6	~~0.~~26.4	literatures [45]

CS: cross-sectional survey, this study conducted a cross-sectional survey in 2011. We conducted a stratified random sampling method to select 12 clinics, located in nine cities (Guangzhou, Foshan, Jiangmen, Qingyuan, Zhaoqing, Dongguan, Shenzhen, Zhuhai, Yangjiang) in Guangdong province. A total of 802 participants were included in this study. The study design has been published elsewhere[46].

Cohort: We established a seven-year cohort study including 14 MMT clinics in Guangdong province. The cohort study recruited 9412 MMT clients between July 2006 and March 2014. The study design has been published elsewhere[47].

域代码已更改

域代码已更改

Table S2 system dynamic equations of the MMT system dynamic model

sub-sytem	type of equation	variables	descriptions of variables	system dynamics equation
MMT Clinic	constant	constant of close	proportion of clinic closed in 2006-2013 (%)	0
	auxiliary	<Time>	time	2006-2013
	constant	MMT CLINIC (initial)	initial number of MMT clinics in 2006	0
	flow	establish rate	annual number of new MMT clinics	IF THEN ELSE(Time<=2007, 10 , IF THEN ELSE(Time<=2008, 3,0))
	flow	close rate	annual number of MMT clinics closed	MMT CLINIC*constant of close
	stock	MMT CLINIC	number of MMT clinics	(+establish rate-close rate, 0)
MMT participants	constant	number of new drug users who have the intention to treat in MMT	number of new drug users who have the intention to treat in MMT	600
	constant	daily cost of MMT	daily cost of MMT (RMB)	20
	auxiliary	time to MMT	routine time to MMT clinics	5×distance to MMT-10

带格式的: 英语(美国)

	constant	distance to MMT	distance to MMT clinics (kilometers)	5
	constant	misconception to MMT	proportion of misconception towards MMT (%)	~~0.~~98
	constant	daily dosage of methadone	average daily dosage of methadone (ml)	50
	constant	proportion of injecting among MMT participants who use drugs	proportion of injecting among MMT participants who use drugs (%)	~~0.~~60
	constant	proportion of drug use among MMT participants	proportion of drug use among MMT participants (%)	~~0.~~20
	constant	Sharing among injecting drug users	Proportion of needle/syringe sharing among injecting drug users (%)	~~0.~~30
	auxiliary	new participants	annual new motivated MMT participants	0.2× number of drug users with intention/motivation to participate MMT ×motivation
	auxiliary	satisfaction	satisfaction factor	90+MMT RETENTION/1300*(-10)

带格式的: 英语(美国)

带格式的: 英语(美国)

	auxiliary	motivation	Proportion of participants who have motivation to participate in MMT	MAX(3.8385-0.044*daily cost of MMT-0.0129*MIN(time to MMT,240)-0.0235*3*MIN(distance to MMT, 60),0)
	auxiliary	injection	proportion of injecting drug users among MMT participants	proportion of injecting among MMT participants who use drugs*proportion of drug use among MMT participants
	auxiliary	Pshare	proportion of needle/syringe sharing among MMT participants	injection*proportion of needle sharing among injecting drug users
	auxiliary	arrest	proportion of being arrested among MMT participants	MIN(proportion of drug use among MMT participants*arrest among drug users , 1)
	auxiliary	drop out	proportion of drop-out	MIN(EXP(-1.5562+0.0124*time to MMT+0.04*daily cost of MMT-0.00577*satisfaction+0.4024*(8*1e-008*distance to MMT*distance to MMT*distance to MMT-2*1e-005*distance to MMT*distance to MMT+0.0027*distance to MMT+0.863)-0.00208*daily dosage of methadone)/(1+EXP(-1.5562+0.0124*MIN(time to MMT,240)+0.04*daily cost of MMT-0.00577*satisfaction+0.4024*(8*1e-008*distance to MMT*distance to MMT*distance to MMT-2*1e-005*distance to MMT*distance to MMT+0.0027*distance to MMT+0.863)-0.00208*daily dosage of methadone))+proportion of drug use among MMT participants*0.2+arrest+misconception to MMT*0.18, 1)

	flow	new enrollment	annual new MMT participants	(DELAY1(establish rate*100 , 1)+new participants,0)
	flow	re-enrollment	annual MMT participants who were re-enrolled	motivation*(drop out*0.85+DELAY1(referral, 0.5))/2.4125
	constant	proportion of successfully detoxification	proportion of successfully detoxification (%)	~~0.0~~0.1
	flow	other detox	annual number of MMT participants successfully detoxed	MMT RETENTION*proportion of successful detoxification
	flow	proportion of deaths	proportion of deaths among MMT participants	DELAY1(0.0286,5)
	flow	die	annual number of deaths among MMT participants	MMT RETENTION*proportion of deaths
	flow	drop out	annual number of drop-outs among MMT participants	MMT RETENTION*proportion of drop out
	stock	MMT RETENTION	number of MMT participants	（INTEGER(new enrollment+"re-enrollment"-die-other detox-drop out)，605）
Detoxification center	constant	Proportion of individuals referred from detoxification center to MMT	Proportion of individuals referred from detoxification center to MMT (%)	~~0.~~20

	auxiliary	referral	annual number of drug users referred to MMT from detoxification center	release rate*Proportion of individuals referred from detoxification center to MMT
	constant	arrest	proportion of MMT participants arrested by the police (%)	~~0.~~20
	flow	annual	number of MMT participants being arrested into detoxification center	proportion of drug use among MMT participants*MMT RETENTION*arrest
	flow	release rate	number of people released from the prison	DELAY1(DC CENTER*0.9,2)
	stock	DC CENTER	number of people in detoxification center	(INTEGER(arrest rate-release rate)，0)
HIV transmission	constant	p HIV(m→f)	the probability of HIV transmission from HIV positive male to HIV negative female through sexual behaviors	0.00124
	constant	Pc	proportion of unprotected sexual behaviors (%)	~~0.~~75
	constant	Ec HIV	effectiveness of condom in protecting from HIV transmission	0.8
	constant	M	proportion of male participants in MMT (%)	~~0.~~90

	constant	HIV prevalence among female drug users in MMT	HIV prevalence among female drug users in MMT (%)	~~0.0~~8
	constant	p HIV(f→m)	the probability of HIV transmission from HIV positive female to HIV negative male through sexual behaviors	0.00377
	constant	F	proportion of female participants in MMT (%)	~~0.~~10
	constant	Pneedle HIV	the probability of HIV transmission per injecting act	0.01
	constant	Pn	the proportion of needle sterilization used by MMT participants when injecting (%)	~~0.~~40
	constant	Pclean HIV	the proportion of being successful sterilized for HIV (%)	~~0.~~70
	auxiliary	Tm	average number of sexual acts per year for male participants	WITH LOOKUP (Time, ([(2004,0)-(2013,200)],(2004,180),(2005,180),(2006,180),(2007,180),(2008,180),(2009,180),(2010,180),(2011,180),(2012,180),(2013,180)))
	constant	p HIV(m→m)	the probability of HIV transmission from HIV positive male to HIV	0.014

			negative male through sexual behaviors	
	constant	HIV prevalence among MSM	HIV prevalence among MSM (%)	~~0.~~11.6
	auxiliary	<HIV prevalence among male participants>	HIV prevalence among male participants	MIN(HIV cases/(MMT RETENTION*proportion of HIV testing+0.0001),1)
	auxiliary	Tf	average number of sexual acts per year for female participants	WITH LOOKUP(Time,([(2004,0)-(2013,200)],(2004,156),(2005,152),(2006,147),(2007,143),(2008,138),(2009,134),(2010,129),(2011,125),(2012,120),(2013,117))）
	auxiliary	Pf-m HIV	The probability of HIV transmission through sexual behaviors	(1-POWER((1-"p HIV(f→m)"),Tf*Pc)*POWER((1-(1-Ec HIV)*"p HIV(f→m)"),(Tf*(1-Pc))))*HIV prevalence among female drug users in MMT
	auxiliary	Pshare	<proportion of needle/syringe sharing among MMT participants>	injection*proportion of needle sharing among injecting drug users
	auxiliary	Tinj	average times of injecting behaviors per year	WITH LOOKUP(Time, ([(2004,0)-(2013,200)],(2004,104.98),(2005,86.51),(2006,101.79),(2007,95.01),(2008,89.96),(2009,83.43),(2010,80.49),(2011,78.05),(2012,76.68),(2013,73.99)))
	auxiliary	Ps HIV	the proportion of HIV transmission through injecting using sterilized	1-POWER((1-Pneedle HIV*(1-Pclean HIV)),Pn*Tinj*Pshare)

			needle/syringe among MMT participants	
	auxiliary	Pu HIV	the proportion of HIV transmission through injecting using unsterilized needle/syringe among MMT participants	1-POWER((1-Pneedle HIV),Tinj*Pshare*(1-Pn))
	auxiliary	Pinj HIV	probability of HIV transmission through injecting behaviors	(1-(1-Ps HIV)*(1-Pu HIV))*Phiv IDU
	auxiliary	Phiv IDU	HIV prevalence among injecting drug users (%)	~~0.~~10
	auxiliary	HIV incidence	HIV incidence among MMT participants	1-(1-Pinj HIV)*(1-Psex HIV)
	constant	p HCV(m→f)	the probability of HCV transmission from HCV positive male to HCV negative female through sexual behaviors	0.00125
HCV transmission	auxiliary	HCV prevalence among MMT participants	HCV prevalence among MMT participants	HCV cases/(MMT RETENTION*proportion of HCV testing)
	constant	HCV prevalence among female drug users in MMT	HCV prevalence among female drug users in MMT (%)	~~0.0~~1.6

	auxiliary	Tm	< average number of sexual acts per year for male participants >	WITH LOOKUP(Time, ([(2004,0)-(2013,200)],(2004,180),(2005,180),(2006,180),(2007,180),(2008,180),(2009,180),(2010,180),(2011,180),(2012,180),(2013,180)))
	constant	Pc	< proportion of unprotected sexual behaviors > (%)	~~0.~~75
	auxiliary	Tf	< average number of sexual acts per year for female participants >	WITH LOOKUP(Time,([(2004,0)-(2013,200)],(2004,156),(2005,152),(2006,147),(2007,143),(2008,138),(2009,134),(2010,129),(2011,125),(2012,120),(2013,117)))
	constant	M	< proportion of male participants in MMT > (%)	~~0.~~90
	constant	F	< proportion of female participants in MMT > (%)	~~0.~~10
	constant	p HCV(f→m)	the probability of HCV transmission from HCV positive female to HCV negative male through sexual behaviors	0.00125
	constant	Ec HCV	effectiveness of condom in protecting from HCV transmission	0.8

	auxiliary	Pf-m HCV	the probability of HCV transmission through sexual behaviors	(1-POWER((1-"p HCV(f→m)"),Tf*Pc)*POWER((1-(1-Ec HCV)*"p HCV(f→m)"),(Tf*(1-Pc))))*HCV prevalence among female drug users in MMT
	constant	p HCV(m→m)	the probability of HCV transmission from HCV positive male to HCV negative male through sexual behaviors	0.0014
	constant	HCV prevalence among MSM	HCV prevalence among MSM (%)	~~0.0~~1.2
	auxiliary	Pshare	proportion of needle/syringe sharing among MMT participants	proportion of injecting among drug users× proportion of needle/syringe sharing among injecting drug users
	constant	Pneedle HCV	The probability of HCV transmission per injecting act	0.03
	constant	Phcv IDU	HCV prevalence among injecting drug users (%)	~~0.~~80
	auxiliary	Ps HCV	the proportion of HCV transmission through injecting using sterilized needle/syringe among MMT participants	1-POWER((1-Pneedle HCV*(1-Pclean HCV)),Pn*Tinj*Pshare)
	auxiliary	Pu HCV	the proportion of HCV transmission through injecting	1-POWER((1-Pneedle HCV),Tinj*Pshare*(1-Pn))

			using unsterilized needle/syringe among MMT participants	
	auxiliary	Pinj HCV	the probability of HCV transmission through injecting behaviors	(1-(1-Ps HCV)*(1-Pu HCV))*Phcv IDU
	auxiliary	HCV incidence	HCV incidence among MMT participants	DELAY1(1-(1-Pinj HCV)*(1-Psex HCV), 1)
	constant	Pn	< the proportion of needle sterilization used by MMT participants when injecting > (%)	~~0.~~40
	constant	Pclean HCV	the proportion of being successful sterilized for HCV (%)	~~0.~~35
HIV testing and treatment system	auxiliary	HIV incidence	<HIV incidence among MMT participants>	1-(1-Pinj HIV)*(1-Psex HIV)
	flow	new infection rate	annual number of new infections among MMT participants	MMT RETENTION*HIV incidence
	stock	UNDIAGNOSED HIV	number of MMT participants who were undiagnosed of HIV	(INTEGER(new infection rate+new HIV positive rate-HIV testing rate-"drop-out rate 1"), 0)
	auxiliary	total proportion of drop-outs among MMT participants	total proportion of drop out	proportion of deaths+proportion of successful detoxification+proportion of drop out

	flow	drop out rate	annual number of MMT participants who dropped out of MMT	total proportion of drop out*UNDIAGNOSED HIV
	auxiliary	Proportion of HIV testing	the proportion of HIV testing among participants in MMT clinics (%)	~~0.~~80
	flow	HIV testing rate	annual number of HIV new testing participants in MMT clinics	UNDIAGNOSED HIV*proportion of HIV testing
	stock	DIAGNOSED HIV	number of diagnosed HIV participants in MMT clinics	(INTEGER(ART drop out rate+HIV testing rate-HIV progress rate-"drop-out rate 2"),155)
	auxiliary	proportion of new HIV positive	the proportion of new HIV positive participants in MMT clinics (%)	~~0.~~10.8
	flow	new HIV positive rate	annual number of new HIV positive participants in MMT clinics	proportion of new HIV positive*enrollment
	constant	proportion of progression to AIDS	the proportion of HIV progression to AIDS stage (%)	~~0.~~25
	flow	HIV progress rate	annual number of new HIV infected participants progress to AIDS in MMT clinics	proportion of progression to AIDS*DIAGNOSED HIV

	stock	AIDS	number of AIDS patients in MMT clinics	(HIV progress rate-"drop-out rate 3"-ART rate,0)
	auxiliary	proportion of progression to AIDS	the proportion of MMT participants in ART	WITH LOOKUP (Time, ([(2004,0)-(2020,10)],(2004,0),(2005,0),(2006,0.01),(2007,0.129),(2008,0.227),(2009,0.298),(2010,0.371),(2011,0.521),(2012,0.6),(2013,0.65),(2014,0.65),(2015,0.65),(2016,0.65),(2017,0.65),(2018,0.65),(2019,0.65),(2020,0.65)))
	flow	ART rate	annual number of MMT participants in ART	AIDS*proportion of participants referred to ART
	auxiliary	HIV cases	accumulated number of HIV infections in MMT clinics	(ART+DIAGNOSED HIV+AIDS,0)
	stock	ART	number of MMT participants in ART	(INTEGER(ART rate-ART drop out rate-HIV deaths rate 4-drop out rate 4),0)
	constant	proportion of drop-outs in ART	the proportion of MMT participants dropping out of ART (%)	~~0.~~25
	flow	ART drop out rate	annual number of MMT participants dropping out of ART	ART*"proportion of drop-outs in ART"
	constant	proportion of deaths for HIV	proportion of MMT participants died of AIDS after initiating ART	DELAY1(0.73,10)

	auxiliary	HIV prevalence among MMT participants	HIV prevalence among MMT participants	HIV cases/(MMT RETENTION*proportion of HIV testing)
HCV testing and treatment	auxiliary	HCV incidence	<HCV incidence among MMT participants>	DELAY1(1-(1-Pinj HCV)*(1-Psex HCV), 1)
	flow	new HCV infection rate	annual new HCV infections among MMT participants	HCV incidence*MMT RETENTION
	stock	UNDIAGNOSED HCV	number of undiagnosed HCV participants in MMT	(new HCV positive rate+new HCV infection rate+new HCV positive rate-"drop-outs rate 5"-HCV clearance among undiagnosed HCV posititve clients-HCV testing rate,1050)
	auxiliary	proportion of HCV testing	the proportion of HCV testing among participants in MMT clinics (%)	0.80
	flow	HCV testing rate	annual number of HCV new testing participants in MMT clinics	UNDIAGNOSED HCV*proportion of HCV testing
	auxiliary	proportion of new HCV positive	The proportion of new HCV positive participants in MMT clinics (%)	0.80
	flow	new HCV positive rate	annual number of new HCV positive participants in MMT clinics	proportion of new HCV positive*enrollment

	stock	DIAGNOSED HCV	number of HCV diagnosed HCV participants in MMT clinics	(+HCV testing rate+HCV treatment drop out rate-"drop-outs rate 6"-HCV clearance among diagnosed HCV-HCV treatment rate,299)
	auxiliary	proportion of HCV treatment	the proportion of MMT participants receiving HCV treatment	WITH LOOKUP (Time, ([(2006,0)-(2020,10)],(2006,0.2),(2007,0.2),(2008,0.2),(2009,0.2),(2010,0.2),(2011,0.5),(2012,0.6),(2013,0.6),(2014,0.6),(2015,0.6),(2016,0.6),(2017,0.6),(2018,0.6),(2019,0.6),(2020,0.6)))
	flow	HCV treatment rate	annual number of MMT participants receiving HCV treatment	proportion of HCV treatment*DIAGNOSED HCV
	stock	HCV TREATMENT	number of MMT participants have ever received HCV treatment	(HCV treatment rate-"drop-outs rate 7"-cure rate for HCV treatment-HCV treatment drop out rate,0)
	constant	HCV treatment drop-outs	the proportion of MMT participants dropping out of HCV treatment (%)	~~0.~~24
	flow	HCV treatment drop out rate	annual number of MMT participants dropping out of HCV treatment	HCV TREATMENT*"HCV treatment drop-outs"
	constant	Proportion of participants cured for HCV	the proportion of MMT participants cured of HCV after treatment (%)	~~0.~~62

	flow	cure rate for HCV treatment	annual number of MMT participants cured of HCV after treatment	the proportion of MMT participants cured of HCV after treatment × number of MMT participants have ever received HCV treatment
	stock	CURE FOR HCV	number of MMT participants who were ever cured of HCV	(+cure rate for HCV treatment+cure rate due to HCV clearance-"drop-outs rate 9",0)
	auxiliary	HCV cases	accumulated number of HCV infections in MMT clinics	HCV TREATMENT+DIAGNOSED HCV
	auxiliary	HCV prevalence among MMT participants	HCV prevalence among MMT participants	HCV cases/(MMT RETENTION*proportion of HCV testing)
	constant	Proportion of clearance 3	Proportion of participants cured due to self-clearance of HCV (%)	100
	flow	cure rate due to HCV clearance	annual number of MMT participants who were cured due to self-clearance of HCV	HCV CLEARANCE*proportion of clearance3
	stock	HCV CLEARANCE	number of MMT participants who ever being self-clearance of HCV	(+HCV clearance among undiagnosed HCV posititve clients+HCV clearance among diagnosed HCV-"drop-outs rate 8"-cure rate due to HCV clearance,0)
	constant	proportion of clearance 1	the proportion of self-clearance among diagnosed HCV patients in MMT clinics (%)	0.13

	flow	HCV clearance among diagnosed HCV	annual number of HCV self-clearance among diagnosed HCV patients in MMT clinics	proportion of clearance 1*DIAGNOSED HCV
	constant	proportion of clearance 2	the proportion of self-clearance among HCV infected without testing in MMT clinics (%)	~~0.~~13
	flow	HCV clearance among undiagnosed HCV positive clients	annual number of HCV self-clearance among HCV infected without HCV testing in MMT clinics	the proportion of self-clearance among HCV infected without testing in MMT clinics × number of HCV infected without HIV testing

Table S3 Effectiveness of health education, psychological counseling, contingency management, needle exchange program, condom promotion, ART and HCV treatment

Interventions	Misconception (%)	drug use (%)	injecting drug use (%)	needle sharing (%)	unprotected sex (%)	ART (%)	HCV treatment (%)	Source
Origin	0.98	0.20	0.60	0.30	0.75	0.60	0.40	
Health Education	0.78	--	--	0.25	--	--	--	Meta-analysis[48]
Psychological Counseling	--	0.11	0.25	0.19	--	--	--	Literatures[49, 50]
Contingency Management	--	0.17	0.24	0.21	--	--	--	Literatures[50, 51]
Needle Exchange Program	0.57	--	--	0.23	0.63	--	--	Meta-analysis[52]
Condom Promotion	--	--	--	--	0.30	--	--	Literatures[53]
ART	--	--	--	--	--	0.75	--	Literatures[44]
HCV Treatment	--	--	--	--	--	--	0.78	Literatures[54]

带格式表格

Reference

1. Zhang L, Zou X, Zhang D, Li X, Zhao P, Ling L. Investigation of Repeat Client Drop-Out and Re-Enrolment Cycles in Fourteen Methadone Maintenance Treatment Clinics in Guangdong, China. PloS one 2015;10:e0139942.
2. Li X. Study on the relationship between the patients' accessibility and treatment outcome in methadone maintenance treatment in Guangdong Province.School of Public Health, Guangzhou: Sun Yat-sen University; 2015, p. 86.
3. Gu J, Xu H, Lau JT, Hao Y, Zhong Y, Fan L, Zhao Y, Hao C, Ling W. Misconceptions predict dropout and poor adherence prospectively among newly admitted first-time methadone maintenance treatment clients in Guangzhou, China. Addiction (Abingdon, England) 2012;107:1641-9.
4. Chen W, Xia Y, Hong Y, Hall BJ, Ling L. Predictors of continued HIV-risk behaviors among drug users in methadone maintenance therapy program in China--a prospective study. Harm reduction journal 2013;10:23.
5. Meng J, Burris S. The role of the Chinese police in methadone maintenance therapy: a literature review. The International journal on drug policy 2013;24:e25-34.
6. Ma Y, Du C, Cai T, Han Q, Yuan H, Luo T, Ren G, Mburu G, Wang B, Golichenko O, Zhang C. Barriers to community-based drug dependence treatment: implications for police roles, collaborations and performance indicators. Journal of the International AIDS Society 2016;19:20879.
7. Chen J, Chen W, Ling L, Xia Y, He Q, Chen A, Han L. Dosage Using in Community Methadone Maintenance Treatment at Clinics in Guangdong and Its Evaluation. Modern Preventive Medicine 2010;37:1687-9.
8. Yan X, Yang Y, Gong J, Min J, Li G. The Methadone Dosage on Methadone Maintain Treatment in Jiangxi Province and Its Influencing Factors.Forum on Low-Carbon Life and Harm on Health, Inner Mongolia: China Environment Mutagen Society of Risk Assessment Committee; 2011, pp. 105-15.
9. Zhang L, Chow EP, Zhuang X, Liang Y, Wang Y, Tang C, Ling L, Tucker JD, Wilson DP. Methadone maintenance treatment participant retention and behavioural effectiveness in China: a systematic review and meta-analysis. PloS one 2013;8:e68906.
10. Luo X, Zhao P, Gong X, Zhang L, Tang W, Zou X, Chen W, Ling L. Concurrent Heroin Use and Correlates among Methadone Maintenance Treatment Clients: A 12-Month Follow-up Study in Guangdong Province, China. International journal of environmental research and public health 2016;13.

11. Marienfeld C, Liu P, Wang X, Schottenfeld R, Zhou W, Chawarski MC. Evaluation of an implementation of methadone maintenance treatment in China. Drug and alcohol dependence 2015;157:60-7.

12. Newspaper BT. Why the proportion of detoxification is lower than 2%? Beijing: Beijing Techonology Newspaper; 2005.

13. Zhou T, Fu Z, Wu Y, Zhu X, Wang J. Establishment and Practice of the Referral Mechanism from Compulsory Detoxification Agencies to Methadone Maintenance Treatment Clinic. Chinese Journal of ADIS and STDs 2011;17:73-5.

14. Wang G, Lin F, Chang D, Song L, Wang W. Referral Mechanism Between Compulsory Detoxification Center to Methadone Maintenance Treatment Clinics in Some City. Modern Preventive Medicine 2010;37:2344-7.

15. Boily MC, Baggaley RF, Wang L, Masse B, White RG, Hayes RJ, Alary M. Heterosexual risk of HIV-1 infection per sexual act: systematic review and meta-analysis of observational studies. The Lancet Infectious diseases 2009;9:118-29.

16. Baggaley RF, White RG, Boily MC. HIV transmission risk through anal intercourse: systematic review, meta-analysis and implications for HIV prevention. International journal of epidemiology 2010;39:1048-63.

17. Everhart JE, Di Bisceglie AM, Murray LM, Alter HJ, Melpolder JJ, Kuo G, Hoofnagle JH. Risk for non-A, non-B (type C) hepatitis through sexual or household contact with chronic carriers. Annals of internal medicine 1990;112:544-5.

18. Sexual Transmission of HCV. http://www.hepcassoc.org/news/article27.html. In: Association HC, editor.; 2002.

19. Chow EPF, Tucker JD, Wong FY, Nehl EJ, Wang Y, Zhuang X, Zhang L. Disparities and risks of sexually transmissible infections among men who have sex with men in China: a meta-analysis and data synthesis. PloS one 2014;9:e89959. doi: 10.1371/journal.pone.0089959.

20. Liao Y, Jiang J, Wei S, Li M, Shi S, He B, Xiang S, Deng W, Ning C, Xu N, Wei F, Liang B, Wei B, Liang H. Changes in Sexual Behaviors After Drug Use and Their Influencing Factors Among Male Drug Users in Guangxi, China. Chinese Journal of Disease Control and Prevention 2012;16:1032-5.

21. He Y, Ruan Y, Teng T, Hao Q, Qin G, Wu J, Zhu G, Chen K, Liu S, Zhou F, Shao Y. Drug use Behaviors and Sexual Characteristics among Community-based Female Intravenous Drug users. Chinese Journal of ADIS and STDs 2003;9:343-6.

22. Health CMo, UNAIDS, WHO. 2011 Estimates for the HIV/AIDS Epidemics in China. Beijing; 2011.

域代码已更改

23. Zhuang X, Liang Y, Chow EP, Wang Y, Wilson DP, Zhang L. HIV and HCV prevalence among entrants to methadone maintenance treatment clinics in China: a systematic review and meta-analysis. BMC infectious diseases 2012;12:130.

24. Li YZ, Xu JJ, Qian HZ, You BX, Zhang J, Zhang JM, Hu QH, Chu ZX, Liu SY, Jiang YJ, Geng WQ, Shang H. High prevalence of HIV infection and unprotected anal intercourse among older men who have sex with men in China: a systematic review and meta-analysis. BMC infectious diseases 2014;14:531.

25. Qian HZ, Yang Z, Shi X, Gao J, Xu C, Wang L, Zhou K, Cui Y, Zheng X, Wu Z, Lu F, Lai S, Vermund SH, Shao Y, Wang N. Hepatitis C virus infection in former commercial plasma/blood donors in rural Shanxi Province, China: the China Integrated Programs for Research on AIDS. The Journal of infectious diseases 2005;192:1694-700.

26. Pinkerton SD, Abramson PR. Effectiveness of condoms in preventing HIV transmission. Social science & medicine (1982) 1997;44:1303-12.

27. Smith DK, Herbst JH, Zhang X, Rose CE. Condom effectiveness for HIV prevention by consistency of use among men who have sex with men in the United States. Journal of acquired immune deficiency syndromes (1999) 2015;68:337-44.

28. Liu H, Su Y, Zhu L, Xing J, Wu J, Wang N. Effectiveness of ART and condom use for prevention of sexual HIV transmission in serodiscordant couples: a systematic review and meta-analysis. PloS one 2014;9:e111175.

29. Baggaley RF, Boily MC, White RG, Alary M. Risk of HIV-1 transmission for parenteral exposure and blood transfusion: a systematic review and meta-analysis. AIDS (London, England) 2006;20:805-12.

30. Gerberding JL. Management of occupational exposures to blood-borne viruses. The New England journal of medicine 1995;332:444-51.

31. UNAIDS. 2008 Report on the Global AIDS Epidemic. New York; 2008.

32. Cheng F, Chen H, Li J, Zhang L, Hu H, Zhang J, Cheng H, Stewart W, Shen J. Sash Survey on High Risk Behaviors of IDUs in Four Cities of Yunnan and Sichuan. Chinese Journal of Drug Dependence 2003;12:294-8.

33. Abdala N, Gleghorn AA, Carney JM, Heimer R. Can HIV-1-contaminated syringes be disinfected? Implications for transmission among injection drug users. Journal of acquired immune deficiency syndromes (1999) 2001;28:487-94.

34. Hagan H, Thiede H. Does bleach disinfection of syringes help prevent hepatitis C virus transmission? Epidemiology (Cambridge, Mass) 2003;14:628-9; author reply 9.

35. Agolini G, Russo A, Clementi M. Effect of phenolic and chlorine disinfectants on hepatitis C virus binding and infectivity. American journal of infection control 1999;27:236-9.

36. Li X. A Research of the Incidence of HIV/HCV/HBV/Syphilis and Risk Behaviors Among Injecting Drug Users in Xichang County of Sichuan Province.Epidemiology and Medical Statistics, Suzhou: Soochow University; 2011.

37. Xia X, Luo J, Bai J, Yu R. Epidemiology of hepatitis C virus infection among injection drug users in China: systematic review and meta-analysis. Public health 2008;122:990-1003.

38. Micallef JM, Kaldor JM, Dore GJ. Spontaneous viral clearance following acute hepatitis C infection: a systematic review of longitudinal studies. Journal of viral hepatitis 2006;13:34-41.

39. van Vlerken LG, Lieveld FI, van Meer S, Koek GH, van Nieuwkerk KM, Friederich P, Arends JE, Siersema PD, Burger DM, van Erpecum KJ. Adherence to ribavirin in chronic hepatitis C patients on antiviral treatment: Results from a randomized controlled trial using real-time medication monitoring. Clinics and research in hepatology and gastroenterology 2016;40:622-30.

40. Yang Z, Lu Y, Xu Q, Chen X. Virologic responses and tolerance of peginterferon alfa plus ribavirin treatment for patients with chronic hepatitis C virus infection in different age categories. Nigerian journal of clinical practice 2016;19:133-9.

41. Malnick SD, Basevitch A. Peginterferon alfa-2b therapy in acute hepatitis C: impact of onset of therapy on sustained virologic response. Gastroenterology 2006;131:683; author reply -4.

42. Xia YH, Chen W, Tucker JD, Wang C, Ling L. HIV and hepatitis C virus test uptake at methadone clinics in Southern China: opportunities for expanding detection of bloodborne infections. BMC public health 2013;13:899.

43. Huan Z, Fuzhi W, Lu L, Min Z, Xingzhi C, Shiyang J. Comparisons of Adherence to Antiretroviral Therapy in a High-Risk Population in China: A Systematic Review and Meta-Analysis. PloS one 2016;11:e0146659.

44. Zhao Y, Shi CX, McGoogan JM, Rou K, Zhang F, Wu Z. Predictors of accessing antiretroviral therapy among HIV-positive drug users in China's National Methadone Maintenance Treatment Programme. Addiction (Abingdon, England) 2015;110 Suppl 1:40-50.

45. Mellors JW, Munoz A, Giorgi JV, Margolick JB, Tassoni CJ, Gupta P, Kingsley LA, Todd JA, Saah AJ, Detels R, Phair JP, Rinaldo CR, Jr. Plasma viral load and CD4+ lymphocytes as prognostic markers of HIV-1 infection. Annals of internal medicine 1997;126:946-54.

46. Deng L, Zou X, Chen W, Xia Y, Liu Y, Ling L. How clients' during-treatment motivations relate to their perceptions and impressions of methadone maintenance treatment: A multilevel analysis of a cross-sectional survey in Guangdong Province, China. Drug and alcohol dependence 2016;164:151-7.

47. Zou X, Ling L, Zhang L. Trends and risk factors for HIV, HCV and syphilis seroconversion among drug users in a methadone maintenance treatment programme in China: a 7-year retrospective cohort study. BMJ open 2015;5:e008162.

48. Liu Z, Qin X, Hu Z, Wu S, Xu X, Li L. The Effectiveness of Health Education on Chinese Drug Users: A Systematic Review and Meta-analysis. Chinese Journal of Health Statistics 2010;27:40-9.

49. Zhang B, Yang L, Wang H, Ji H, Xu Y, Wen Z, Li Y, Li J, Wang C. Effects of Psychological Counseling Intervention on Increasing Methadone Dosage and Reducing Heroin Use Among Patients Receiving Methadone Maintenance Treatment. Chinese Journal of Epidemiology 2015;36:832-5.

50. Chen W. Effectiveness and Health Economics Evaluation on Psychosocial and Behavioral Intervention among Community-based Methadone Maintenance Treatment.Epidemiology and Medical Statistics, Guangzhou: Sun Yat-sen University; 2013, p. 172.

51. Chen W, Hong Y, Zou X, McLaughlin MM, Xia Y, Ling L. Effectiveness of prize-based contingency management in a methadone maintenance program in China. Drug and alcohol dependence 2013;133:270-4.

52. Zhang Y, Tang Q, Wang W, Tan H. Meta-analysis on the Effect of Needle Exchange on Prevention and Control of HIV/AIDS Among Injecting Drug users. Practical Preventive Medicine 2011;18:1406-9.

53. Zhu J, Zhong B, Liu Y, Wang P, Wang S, Yan J, Ouyang S, Fu Z, Chen D, He P. The Preventive Effect of 100% Condom Use Programme (CUP) for Sexually Transmitted Disease Among Outpatients Receiving Methadone Maintenance Treatment. Chinese Journal of Disease Control and Prevention 2012;16:130-2.

54. Harris KA, Jr., Arnsten JH, Litwin AH. Successful integration of hepatitis C evaluation and treatment services with methadone maintenance. Journal of addiction medicine 2010;4:20-6.
